# Urothelial Papilloma of the Urinary Bladder: A Case Report and Literature Review of a Rare Entity

**DOI:** 10.7759/cureus.22046

**Published:** 2022-02-09

**Authors:** Ahmed M Abdel Gawad, Ahmed Rabie, Mohammed S Abdelwahed, Abdulkarim Hasan

**Affiliations:** 1 Urology, Faculty of Medicine, Al-Azhar University, Damietta Branch, New Damietta, EGY; 2 Pathology, Faculty of Medicine, Al-Azhar University, Damietta Branch, New Damietta, EGY; 3 Pathology, Faculty of Medicine, Al-Azhar University, Cairo, EGY; 4 Pathology, Faculty of Medicine, University of Jeddah, Jeddah, SAU

**Keywords:** urothelial carcinoma, urinary bladder, bladder tumors, case report, urothelial papilloma

## Abstract

Urothelial papilloma is a rare benign neoplasm composed of a delicate fibrovascular core covered by normal urothelium. In this report, we present a case of an incidentally detected endovesical papillary growth in ultrasound scan (USS) during a routine check-up, suggestive of bladder tumor which was also suggested by CT urography. The tumor was initially managed with transurethral resection and immediate intravesical instillation of mitomycine C 40 mg. The histopathologic assessment of the specimen concluded that the growth was “urothelial papilloma with no atypia or malignancy.” Although papilloma is unequivocally benign, it can recur; recurrences can be multiple and can occur years after the initial diagnosis occasionally with progression to carcinoma, hence long-term surveillance is essential. Our case was followed up for a year with no signs of recurrence or progression and long-term surveillance will be done annually.

## Introduction

Urothelial papilloma (UP) has very restrictive diagnostic features, where papillary fronds are lined with normal-appearing urothelium in histopathology. Defined as such, it is a rare benign tumor. Its diagnosis is essentially based on light microscopy using architectural and cytologic features with immunohistochemistry playing no major essential role [1].

The rarity of UPs results in any one observer having limited experience because even large institutions often have no more than one case per year [2]. Owing to the rarity of UPs, the objective of this report is to present our incidental finding of a case with an exophytic UP and review the literature for appropriate diagnosis and management.

## Case presentation

A 52-year-old male patient, ex-smoker, presented during a routine medical check-up with an incidentally discovered intravesical growth (20 mm × 7 mm) in ultrasound scan (USS) which was confirmed by CT urography. The patient had no urinary symptoms apart from a history of one attack of gross painless hematuria eight months earlier for which the patient did not seek medical advice as it spontaneously resolved. His past medical and surgical history was uneventful except for a “tubulovillous adenoma” at the rectosigmoid junction after a colonoscopic biopsy two years ago. The physical examination was unremarkable except for morbid obesity (body mass index, BMI: 38.6). Digital rectal examination (DRE) was normal with non-suspicious flat prostate.

Hematological and biochemistry investigations did not reveal any abnormality. His urinalysis was clear (no pyuria or hematuria), and total prostatic specific antigen (PSA) was normal (0.964 ng/mL).

**Figure 1:**
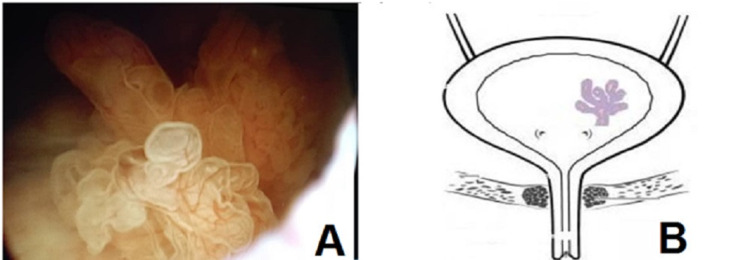
Cystoscopic view. A: Cystoscopic view showing an isolated exophytic pedunculated soft pinkish growth with delicate papillary structures. B: A drawing showing the relation of the growth to the ureteric orifice.

After informed written consent, the patient underwent diagnostic cystourethroscopy and proceeded for transurethral resection of bladder tumor (TUR-BT) on November 14th, 2019 at our institution. Diagnostic cystourethroscopy revealed a normal urethra and an “exophytic papillary soft-tissue growth” in the vicinity of the left ureteric orifice (Figure 1).

Bi-polar TUR-BT was performed with en-bloc resection of exophytic part of the tumor, its underlying muscularis propria, and the edges of the resection area, along with immediate intravesical instillation of mitomycine C 40 mg.

**Figure 2:**
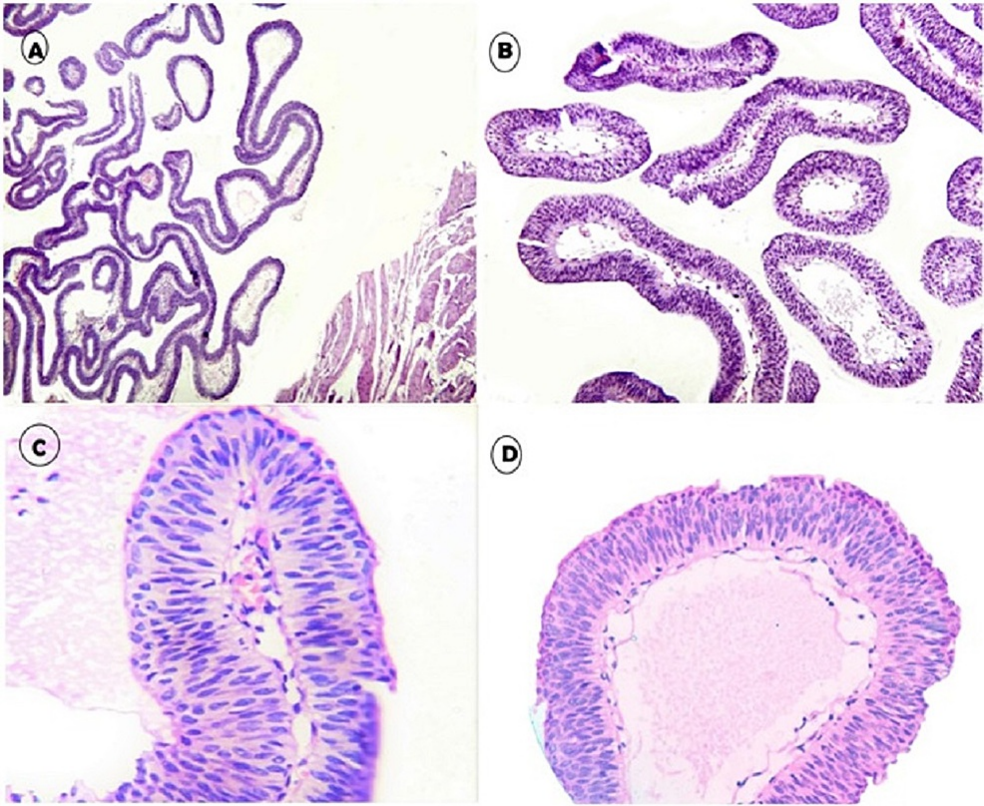
Microscopic picture. The UP of the urinary bladder with: (A) Discrete papillary structures, hierarchical branching without invasion to lamina propria or muscularis propria (H&E, 40x). (B) Slender papillae having fibrovascular cores (H&E, 100x). (C) Fibrovascular cores lined by urothelial cells not exceeding seven to eight layers. The cells show uniform bland oval nuclei and pink cytoplasm without atypia or mitosis (H&E, 400x). (D) Occasional cores showing marked edema, giving it a bulbous look. The lining urothelium appears normal with prominent umbrella cell layer (H&E, 200x). UP, urothelial papilloma

The histopathologic assessment of the specimen concluded that the growth was “urothelial papilloma with no signs of atypia or malignancy” (Figure 2).

A second look cystoscopy with a sampling of the ex-tumor site was performed at three months. There were no signs of recurrence and the sample was benign with just reactive changes of the urothelium.

Owing to its uncertain biologic potential, and since the duration of surveillance is not consensual in the literature [3], we consider following up our patient with urinalysis, USS, and diagnostic cystoscope on an outpatient basis at three, six, and twelve months and then annually. All visits (till November 2021) revealed normal with no signs raising the suspicion for recurrence or progression.

## Discussion

Grading of urothelial tumors has a particular importance, especially in non-invasive papillary neoplasms, which include exophytic UP, papillary urothelial neoplasm of low malignant potential (PUNLMP), low-grade papillary urothelial carcinoma, and high-grade papillary urothelial carcinoma [1].

The aim of this classification has always been to define biologically different lesions with differences in their clinical outcomes. It has distinguished papilloma that is essentially benign, from PUNLMP that poses favorable oncologic outcome and no longer carry the label of “cancer,” from the most malignant; non-invasive high-grade papillary carcinomas that have unfavorable prognosis as they express biologic properties (high level of genetic instability) similar to invasive urothelial carcinomas [4].

While PUNLMP, low-grade papillary carcinoma, and high-grade papillary carcinoma show changes parallel that were seen in hyperplasia, dysplasia, and carcinoma in situ (CIS), respectively, UP is strictly defined as a papillary growth composed of a delicate fibrovascular core covered with urothelium of normal thickness, cellularity, and polarization [1].

It is characterized histologically by discrete papillary fronds, with occasional branching, but without fusion. The stroma may show edema and/or scattered inflammatory cells, the epithelium lacks atypia and superficial (umbrella) cells are often prominent. Mitoses are absent to rare and, if present is basal in location and not abnormal. The lesions may occasionally show a concomitant inverted growth pattern [2, 5]. Rarely, papillomas extensively involve the mucosa; this is referred to as diffuse papillomatosis [5].

Given the above-restricted criteria, the incidence of exophytic UP of the bladder is low; usually 1%-4% of all bladder tumors but it might be even rarer since, in a prospective study conducted over two years period in Western Sweden, of all bladder tumor cases diagnosed, no recorded case of UP among 701 patients [6].

The UPs are commonly but not exclusively seen at a younger age than other urothelial tumors with a male: female ratio of 1.9:1 and often present with hematuria [7].

Endoscopic evaluation is the first step for the diagnosis of unusual cystic lesions including UP [8]. Almost all patients have a single small (<2 cm in greatest dimension) lesion commonly located on the posterior or lateral bladder walls in the vicinity of the ureteric orifices. Since grossly, exophytic UP of the bladder is essentially identical to PUNLMP or low-grade papillary urothelial carcinoma, TUR-BT is the treatment of choice [3, 9].

The UPs typically have a favorable clinical course, nevertheless, studies reveal that UPs can recur; recurrences can be multiple and can occur years after the initial diagnosis occasionally with progression to carcinoma [10].

Since the time of surveillance should be proportional to the risk of recurrence and progression, and given that the data in the literature on predictive features to distinguish papillomas with different risks of recurrence or progression are lacking [11], a program of surveillance varies among authors with some suggesting a two-year follow-up with cystoscopy every six months while others propose clinical examination and ultrasound carrying out at three, six, and twelve months and then annually [3]. Our patient was followed up with urinalysis, USS, and cystoscope at three, six, and twelve months with the second look cystoscope and biopsy of the ex-tumor site performed three months after TUR-BT to confirm complete resection and no local recurrence. Consecutive follow up is going to take place annually.

## Conclusions

This is a report showing a patient with bladder UP by accident while performing a routine check-up. The relevance of this report is how infrequent it is to see such lesions and how accurate histopathology diagnosis is important for such lesions. We have tried to illustrate the scarcity and benign nature of the tumor, as well as the basic axes of any tumor type, namely tumor characteristics, diagnosis, grading system, histologic features, treatment, and prognosis.
